# Effect of the volatile anesthetic agent isoflurane on lateral diffusion of cell membrane proteins

**DOI:** 10.1002/2211-5463.12443

**Published:** 2018-05-23

**Authors:** Junichiro Ono, Satoko Fushimi, Shingo Suzuki, Kiyoshi Ameno, Hiroshi Kinoshita, Gotaro Shirakami, Kazuya Kabayama

**Affiliations:** ^1^ Department of Anesthesiology Faculty of Medicine Kagawa University Kita‐gun Kagawa Japan; ^2^ Department of Anesthesiology KKR Takamatsu Hospital Kagawa Japan; ^3^ Department of Anatomy and Neurobiology Faculty of Medicine Kagawa University Kita‐gun Kagawa Japan; ^4^ Department of Forensic Medicine Faculty of Medicine Kagawa University Kita‐gun Kagawa Japan; ^5^ Department of Chemistry Graduate School of Science Osaka University Toyonaka Osaka Japan

**Keywords:** live cell imaging, membrane fluidity, volatile anesthetic agent

## Abstract

The volatile anesthetic isoflurane (ISO) has previously been shown to increase the fluidity of artificial lipid membranes, but very few studies have used biological cell membranes. Therefore, to investigate whether ISO affects the mobility of membrane proteins, fluorescence‐labeled transferrin receptor (TfR) and glycosylphosphatidylinositol (GPI)‐anchored protein were expressed in human embryonic kidney 293T cells and neural cells and lateral diffusion was examined using fluorescence recovery after photobleaching. Lateral diffusion of the TfR increased with ISO treatment. On the other hand, there was no effect on GPI‐anchored protein. We also used GC/MS to confirm that there was no change in the concentration of ISO due to vaporization during measurement. These results suggest that ISO affects the mobility of transmembrane protein molecules in living cells.

**Abbreviations**
DPPC: dipalmitoylphosphatidylcholine
FRAP: fluorescence recovery after photobleaching
GABA: gamma‐aminobutyric acid
GPI: glycosylphosphatidylinositol
HEK: human embryonic kidney
MAC: minimum alveolar concentration
NMDA: *N*‐nitrosodimethylamine
ROI: region of interest
TfR: transferrin receptor

The full mechanism of action of volatile anesthetics is not yet known. The complete mechanism underlying the action of volatile anesthetics remains unknown. Volatile anesthetic molecules have been considered to act on specific membrane receptors 1 as well as at the membrane–water interface, thereby increasing membrane fluidity. Tsai *et al*. 2 studied the effects of volatile anesthetics on phospholipid (dimyristoylphosphatidylcholine) hydration in a water‐in‐oil reversed micellar system using Fourier transform infrared spectroscopy and found that they decreased the peak of restricted water molecules and increased the peak of free water molecules. In addition, the peak shift of phosphate toward a higher wave implied that water molecules bind to the phosphate moiety of the anesthetic molecules. Hamanaka *et al*. 3 reported the effects of the volatile anesthetics diiodomethane and trifluoroethyl iodide on the purple membrane of *Halobacterium halobium*. X‐ray diffraction profiles indicated that anesthetic molecules were present on the lipid side of the protein–lipid interface. Furthermore, Tang *et al*. 4 proposed that volatile anesthetics preferentially target the lipid–protein–water interface and not a specific channel protein. They simulated the interaction between halothane molecules and the gramicidin A channel using large‐scale 2.2‐ns all‐atom molecular dynamic simulation. Their results suggested that 10 molecules of halothane were distributed between channel protein and dimyristoylphosphatidylcholine membrane. However, the halothane molecules did not penetrate into channel proteins. Yoshida *et al*. 5 analyzed the action of enflurane on a water‐in‐oil (glycerol α‐monooleate/*n*‐decane/water) emulsion using proton nuclear magnetic resonance spectroscopy. They showed that volatile anesthetics weakened the hydrogen bonds of the oil–water interface and desorbed the vicinal water. Ueda *et al*. 6 reported the effects of halothane on surface viscosity using a dipalmitoylphosphatidylcholine (DPPC) monolayer. Halothane decreased the viscosity (increased fluidity) of the DPPC membrane in a dose‐dependent manner. They concluded that the presence of volatile anesthetics at the membrane–water interface dampened lipid–water interaction forces and consequently increased membrane fluidity.

These findings help our understanding of the distribution and effects of anesthetics within the membranes; however, the mechanisms in live cell membranes remain elusive. The living cell membrane comprises heterogeneous lipids. The binding of volatile anesthetics to membrane lipids is closely related to the partition coefficient between anesthetics and lipid molecules. Therefore, the distribution of volatile anesthetics in the biomembrane is predicted to be heterogeneous. We hypothesized that the heterogeneous distribution of anesthetic molecules in living membranes would result in heterogeneous changes in membrane fluidity.

To verify this hypothesis, we investigated the fluidity of marker proteins in the cell membrane using fluorescence recovery after photobleaching (FRAP). HaloTag‐fused transferrin receptor (TfR) and glycosylphosphatidylinositol (GPI) anchor protein, representative markers proteins in the cell membrane, were expressed in human embryonic kidney (HEK) 293T cells and neural cells. These cells were exposed to isoflurane (ISO), a volatile anesthetic generally used in clinical medicine, and were analyzed using FRAP to ascertain whether ISO induced heterogeneous changes in fluidity. We also used midazolam (MDZ), a general anesthetic, water‐soluble benzodiazepine agonist. Unlike volatile anesthetics that act on multiple sites in the cell, MDZ specifically binds to the gamma‐aminobutyric acid A (GABA_A_) receptor. Hence, MDZ was predicted to induce minimal changes in membrane fluidity.

Because volatile anesthetics are easily vaporized at room temperature, we closely monitored the aqueous concentration of ISO during the experiment using GC/MS.

## Materials and methods

### Construction of marker proteins

The two marker proteins were generated by gene synthesis (Genscript, Piscataway, NJ, USA). The protein domains were connected in a pcDNA3 vector (Thermo Fisher Scientific, Waltham, MA, USA) using restriction sites introduced at the end of the synthesized domains. The combinations of the domains from the N terminus to the C terminus were as follows. HaloTag‐TfR (as a type of transmembrane protein): full‐length human TfR, with its stop codon (TAA) replaced by GGG, was fused to HaloTag^®^ domain (Promega, Madison, WI, USA) which covalently bind to HaloTag® ligand (pcDNA3‐TfRHL); HaloTag‐GPI (as a type of lipid‐anchored protein): human thy1‐derived signal sequence from Met1 to Gln20, HaloTag domain, with its around stop codon (CGGACCGTCTAA) replaced by CTG, and human thy1 GPI‐anchoring domain from Asp125 to Leu161 was fused sequentially (pcDNA3‐ssHLGPI). Gene sequences were verified by Sanger sequence. These fused HaloTag domains were expressed in the extracellular region. Whole‐plasmid sequences of pcDNA3‐TfRHL and pcDNA3‐ssHLGPI are described in Fig. S2.

### Cell culture

Human embryonic kidney 293T cells were plated in an 8‐well Nunc Lab‐Tek II chambered coverglass system (Thermo Fisher Scientific) at a density of 1 × 10^4^ cells and grown to subconfluence in Dulbecco's modified Eagle's medium (DMEM) supplemented with 2 mmol·L^−1^ l‐glutamine, 100 U·mL^−1^ penicillin, 0.1 mg·mL^−1^ streptomycin, and 10% FBS.

Primary neural cell cultures from cerebral cortices were prepared from embryonic day 20 (E20) Wistar rats 7. Cerebral cortices were cut into small pieces and incubated at 37 °C for 30 min in papain solution (10 units·mL^−1^ papain in PBS). The neural cells were passed through a 70‐μm cell strainer (Falcon, Oxnard, CA, USA) to remove debris and then plated in a Nunc Lab‐Tek II chambered coverglass system at a final density of 1 × 10^5^ cells and cultured in DMEM containing 5% FBS. The medium was changed to Neurobasal medium (Sigma‐Aldrich, St. Louis, MO, USA) containing B27 supplement and glutamine (Thermo Fisher Scientific) the next day and supplemented with 10 μm cytosine‐β‐d‐arabinofuranoside (Ara‐C) after 3 days. The culture medium was replaced with new culture medium containing Ara‐C every fourth day. Cells were maintained in an incubator with 5% CO_2_ for 10 days.

This study was performed in accordance with protocols approved by the Institutional Animal Care and Use Committee of Kagawa University, Japan (Approval number 15122).

### FRAP analysis

HEK293T and neural cells were transfected with the fusion protein encoding expression plasmids using Lipofectamine 2000 (Thermo Fisher Scientific) 24 h prior to FRAP analysis. The cell‐impermeable ligand (HaloTag Alexa Fluor 488 ligand) was added to the medium 60 min before FRAP analysis to determine specific labeling of cell‐surface proteins, and the culture medium was replaced with 1 mm ISO dissolved in DMEM 30 min before FRAP experiments. The FRAP experiment was performed using a Zeiss LSM710 confocal microscope system with a 63× oil‐immersion objective (Carl Zeiss, Oberkochen, Germany). A 1‐μm‐radius circular region of interest (ROI) was selected on the cell membrane. The imaging size was 512 × 128 pixels, and the scan speed was 33 μs per pixel. The experiments began by obtaining five images to record the prebleach intensity at 0.1% laser power, followed by full laser power photobleaching and a postbleach sequence of 300 images. Fluorescence intensity in ROI was adjusted using background subtraction. The analysis values were calculated with nonlinear regression software, originpro 2015 (Lightstone, Chiyoda‐ku, Tokyo, Japan), using the following equations:

$$y=y_{0}+\sum_{\left(n=1\right)}^{\infty }A_{n}*\text{exp}\left(-x/t_{n}\right)$$

where *y* is the recovery ratio at the arbitrary point of time (*x*), *t*_*n*_ is the time constant, *A*_*n*_ is the constant, and *y*_0_ is a theoretical plateau value for the recovery ratio (the so‐called mobile fraction, *M*_f_). The half‐time to recovery (*t*_1/2_) was determined as:

$$t_{1/2}=t_{n}*\text{ln}\left(2\right)$$

Here, *t*_1/2_ is the time required for half the value of *y*_0_ and depends on the average speed of the marker molecules in ROI. In contrast, *M*_f_ depends on the number of moving molecules in ROI. The larger the number of mobile molecules in ROI, the greater is the value of *y*_0_ (mobile fraction).

### Measurement of isoflurane concentration using GC/MS

Prior to the FRAP experiments, we confirmed the aqueous concentration of ISO in the cell culture medium using GC/MS as previously described, with slight modifications 8. Using a gastight syringe (Hamilton Company, Reno, NV, USA), media samples were collected before and after incubation under the same culture conditions as those used for the FRAP assay. Each sample (100 μL) was immediately placed in ice‐cold glass vials containing 500 μL of *n*‐heptane with 3 mm halothane (internal standard) and sealed with Teflon‐lined caps. After vortexing for 1 min, vials were centrifuged at 800 ***g*** for 3 min. An injection volume of 1 μL of the organic phase was used for GC/MS analysis.

The saturated molar concentration of ISO in DMEM (15 mm) was calculated as previously described 9. In brief, 1 mL of ISO was added to 100 mL of DMEM and stirred overnight in a gastight glass bottle, followed by gravity‐driven phase separation for at least 3 h at room temperature. We freshly prepared three different dilutions (1 : 1, 1 : 3, and 1 : 9, resulting in 7.5, 3.75, and 1.5 mm ISO, respectively) from the saturated ISO solution and used these for GC/MS analysis. A linear calibration curve was obtained from the peak area ratio of ISO /halothane ranging from 1.5 to 15 mm (*Y* = 87.427*X* − 0.1566; *R* ^2^ = 0.9997).

### Statistical analysis

All numerical values were expressed as mean ± standard deviation. Statistical significance was determined by Student's *t*‐test, and *P*‐values < 0.05 were considered statistically significant.

## Results

### Treatment of cells with isoflurane and midazolam

**Figure 1:**
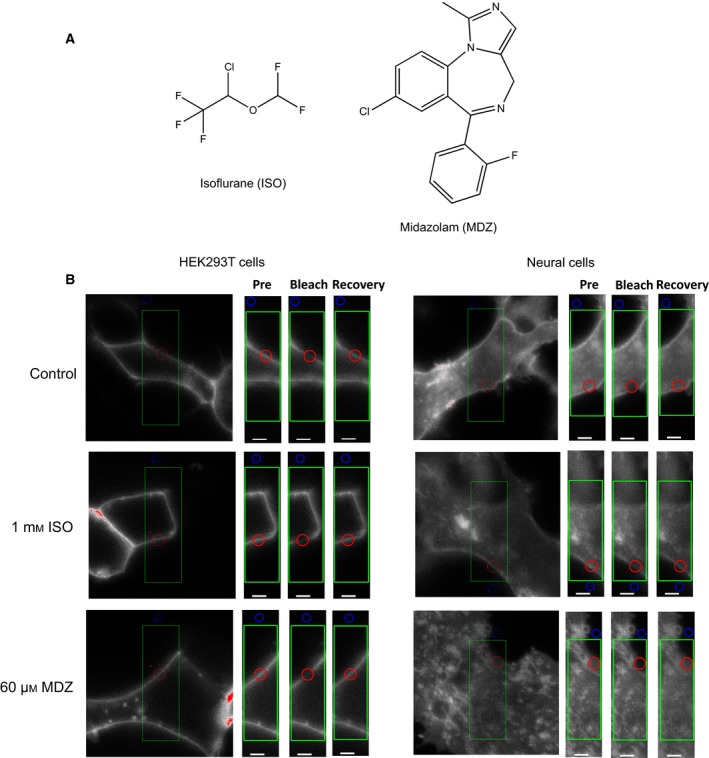
Anesthetic agents and cell morphology before and after anesthesia. (A) Chemical structure of ISO and MDZ. (B) Cell morphology before and after anesthesia (1 mm ISO, or 60 μm MDZ, 30 min) in FRAP analysis. The cDNA plasmids of the HaloTag‐TfR were transiently transfected into each cell. After 1 day, 1 μm cell membrane impermeability ligand Alexa Fluor®488–HaloTag ligand (Promega) was added, and after 60 min, expression of the fluorescence probe was observed by confocal laser scanning microscopy (LSM710; Zeiss). No change in morphology was observed using FRAP even after anesthetic action in HEK293T cells and neural cells, in any imaging period. Pre, prebleaching; bleach, just after bleaching; recovery, 60 s after bleaching; red circle, bleaching area (ROI); blue circle, background; green square, measurement area. Scale bar: 2 μm.

To investigate the effect of anesthetic drugs on cell membrane fluidity, we analyzed the lateral diffusion of fluorescence‐labeled membrane molecules in the presence of anesthetic drugs using FRAP. FRAP is a method used to analyze the fluidity of target molecules in living cells using a confocal fluorescence microscope. We studied the frequently used anesthetic drugs ISO, a volatile anesthetic drug generally used in clinical medicine with an unknown detailed mechanism of action, and MDZ, a benzodiazepine agonist (Fig. 1A). To analyze cell membrane fluidity, TfR fused to HaloTag protein was used as a membrane molecule. This was transiently expressed in cells and then allowed to bind to Alexa Fluor® 488, a ligand of HaloTag (Alexa488‐TfR). Cells were treated for 30 min with 1 mm ISO and 60 μm MDZ, the optimal blood concentrations for the clinical use of these anesthetics 10, 11, 12, and then observed under a fluorescence microscope. Microscopic analysis showed neither significant morphological changes nor cell death under the conditions used (Fig. 1B). We selected cells with no morphological changes following drug treatment for FRAP analysis.

In addition, dose of 0.5–2 minimum alveolar concentration (MAC; ED50 concentration of volatile anesthetics) is necessary for surgical anesthesia 13. The calculated 1 MAC equivalent of ISO at 25 °C used in this study was 0.51 mm 10, 11. Therefore, it can be assumed that 1 mm ISO (2 MAC) is clinically relevant. We attempted a similar study with 2.5 mm ISO (data not shown). However, as remarkable changes were observed in the cell membrane morphology at this concentration, FRAP analysis was not performed.

### Changes in cell membrane fluidity after anesthetic drug treatment

**Figure 2:**
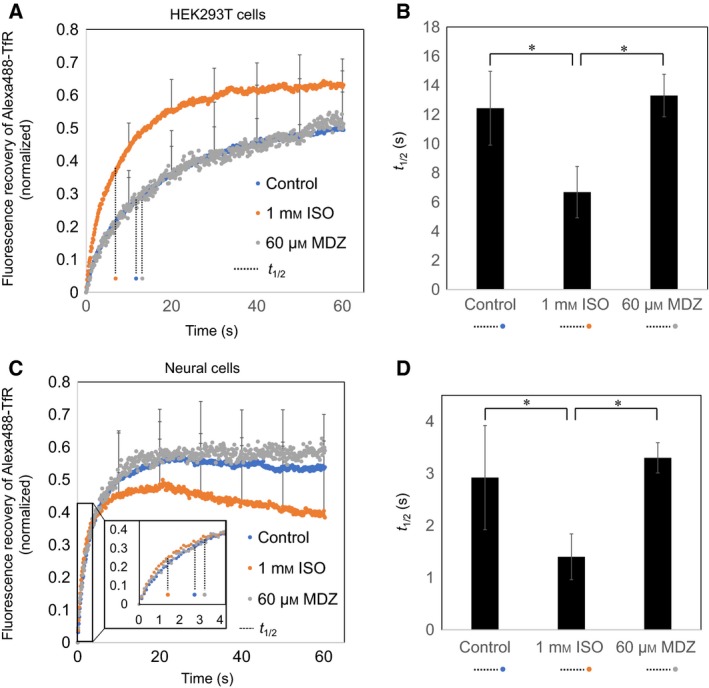
FRAP analysis of HEK293T cells and neural cells. Influence of anesthetic on the recovery of fluorescence intensity in HEK293T cells (A) and neural cells (C). The *t*_1/2_ of fluorescence recovery rate of Alexa488‐TfR in HEK293T cells (B) and neural cells (D) treated with 1 mm ISO for 30 min or 60 μm MDZ for 30 min. Blue circle: control; orange circle: 1 mm ISO; gray circle: 60 μm MDZ; dot line: *t*_1/2_, *P < 0.05 compared with control and MDZ groups.

We analyzed cell membrane fluidity of Alexa488‐TfR by FRAP for 60 s after anesthetic drug treatment. Membrane fluidity measured using FRAP can be explained by two parameters: half‐time to recovery (*t*_1/2_) and mobile fraction (*M*_f_). The *t*_1/2_ is a time constant and is an indicator of the velocity of the moving fluorescence molecules. On the other hand, *M*_f_ is the fluorescence recovery rate in ROI and indicates the number of mobile components. The results obtained by FRAP indicated that MDZ‐treated HEK293T cells were not different compared with the control, whereas ISO‐treated cells showed a significant decrease in *t*_1/2_, indicating an increase in membrane fluidity (Fig. 2A,B). Moreover, we analyzed isolated neural cells using the same FRAP procedure and also found that the *t*_1/2_ of the neural cells decreased significantly, as observed in HEK293T cells (Fig. 2C,D). In HEK293T cells, *M*_f_ increased significantly; however, it decreased in neural cells but not significantly. Furthermore, to investigate whether these changes could occur in other membrane proteins, the same study was conducted using Alexa‐488‐GPI. However, there were no significant changes in either *t*_1/2_ or *M*_f_ values after anesthetic drug treatment (Table 1, Fig. S1). Thus, ISO affects the membrane fluidity of transmembrane proteins in living cells.

**Table 1:** Summary of results of FRAP analysis in anesthetic‐treated HEK293T cells and neural cells

Parameter	Cell	Control	Alexa488‐TfR		Alexa488‐GPI		
			ISO	MDZ	Control	ISO	MDZ
*t*_1/2_	HEK293T cells	12.4 ± 2.53	6.68 ± 1.76^a^	13.3 ± 1.46	3.84 ± 1.07	3.46 ± 1.44	3.43 ± 1.07
	Neural cells	2.92 ± 1.0	1.40 ± 0.44^a^	3.30 ± 0.29	1.69 ± 0.77	1.47 ± 0.65	1.84 ± 0.52
*M*_f_	HEK293T cells	0.51 ± 0.17	0.60 ± 0.10^a^	0.52 ± 0.09	0.75 ± 0.08	0.77 ± 0.14	0.73 ± 0.09
	Neural cells	0.57 ± 0.13	0.44 ± 0.19	0.58 ± 0.01	0.67 ± 0.09	0.65 ± 0.09	0.60 ± 0.08

a *P* < 0.05 compared with control and MDZ groups.

### Quantification of isoflurane in culture media

Because ISO is a volatile molecule and cells were treated with 1 mm saturated ISO solution for 30 min prior to FRAP analysis, it was necessary to confirm that the concentration of ISO in the aqueous phase did not change during the experiment. We prepared eight individual chambers containing various concentrations of ISO solution used in the study. The ISO concentration in each chamber was measured using GC/MS immediately prior to incubation and again after a 30‐min incubation. The saturated concentration of neat ISO, which was theoretically 15 mm, was measured as 13 mm in the chamber, and the concentration was unchanged after 30 min. Furthermore, the measured concentration of the 1 mm ISO solution (diluted in DMEM) stayed the same in the chamber after 30 min; therefore, it was confirmed that the chamber contained appropriate concentrations of ISO. Residual quantities of ISO in 10%, 25%, 50%, and 100% ISO solutions in the culture medium were measured over time by GC/MS and are shown in Table 2.

**Table 2:** Analysis of aqueous concentration of ISO in experimental medium by gas chromatography

	Mean ± standard deviation (mm)
Start, 0 min (n = 4)	
100% (15 mm)	13.37 ± 2.85
50% (7.5 mm)	6.74 ± 0.79
25% (3.75 mm)	3.26 ± 0.62
10% (1.5 mm mm)	1.05 ± 0.36
After 30 min (n = 4)	
100% (15 mm)	13.28 ± 1.59
50% (7.5 mm)	6.00 ± 1.02
25% (3.75 mm)	3.26 ± 0.44
10% (1.5 mm)	1.01 ± 0.20

## Discussion

Isoflurane is a halogenated hydrocarbon. It does not have a specific binding receptor, and its mechanism of action as an anesthetic is unknown; however, a report using artificial lipid membranes showed that it is distributed at the lipid–water interface and weakens the membrane structure 6. ISO enters the lipid membrane of bacteriorhodopsin and acts on the hydrophobic sites of membrane proteins and lipids 3. MDZ, like ISO, is a drug that is clinically used for general anesthesia, and is a benzodiazepine with sedative and antispasmodic actions. Its site of action is the GABA_A_ receptor.

We treated living cells with ISO and MDZ and performed FRAP analysis, a method used for live cell imaging, to observe the dynamics of the GPI anchor and fluorescence‐labeled TfR. We found no significant changes in the MDZ group compared with the control group. Because MDZ acts by binding directly to the GABA_A_ receptor, it is believed that it has little effect not only on lipid membranes but also on the GPI protein TfR. Therefore, it is reasonable that no significant difference was observed in the liquidity of Alexa488‐GPI or Alexa488‐TfR compared with the control group. On the other hand, in the ISO group, the fluidity of Alexa488‐GPI did not change, but that of Alexa488‐TfR did. It has suggested that GPI‐anchored proteins and TfRs exist in different domains on the cell membrane, and ISO may have acted heterologously on these domains. And biochemically, although it is reported that GPI exists in raft domain and TfR exists in nonraft domain, we think that it is difficult to evaluate this difference by FRAP analysis. We assume that at least the volatile anesthetic ISO has a marked influence on the fluidity of the transmembrane protein rather than the membrane lipids. Furthermore, it is also important to consider the influence of the cytoskeleton and its supporting proteins as factors regulating the movement of transmembrane proteins. In particular, the cytoskeleton is thought to limit the diffusion of transmembrane proteins by constructing an actin mesh structure 14. Lenne *et al*. 15 showed that treatment of COS‐7 cells with latrunculin B and cytochalasin D, inhibiting actin polymerization, alleviated restricted transferrin motility brought about by the cytoskeletal network structure. At the same time, they reported that GPI‐anchored proteins were less sensitive to site skeleton agonists (polymerization inhibitors and polymerization stabilizers) than TfR.

A study of nerve cell experiments using volatile anesthetics showed that ISO disturbs the structural stability of the actin cytoskeleton (structural destabilization), inhibits elongation of hippocampal dendritic spines 16, and inhibits the development of immature astrocytes and neurons 17, 18. All of these effects (mainly morphological changes) depend on the anesthetic dose, exposure time, and intracellular actin distribution (dendritic spines are rich in actin). Because the cytoskeleton takes various forms depending on the cell type, the cause of the difference in *M*_f_ change between HEK293T and neural cells observed in our study may be due to characteristics of the cytoskeleton in each cell. In other words, differences in the sensitivity of various cells to ISO may be attributed to the differences in the cytoskeleton between cells. Although the primary cultured cells were used only on the 10th day in this study, the level and composition of sphingolipids in neural cells changes greatly as the cells mature 19, and therefore, membrane fluidity in response to ISO at each stage of differentiation may vary. Age‐ and organ‐dependent differences in anesthesia susceptibility 20 may be due to differences in membrane fluidity as a result of differences in sphingolipid composition. And considering the effects of actin skeleton and sphingolipid composition on the plasma membrane, the difference in time constant of lateral diffusion of Alexa488‐TfR (*t*_1/2_ of HEK293T: 12 s; and *t*_1/2_ of neural cells: 3 s) may be an indicator of anesthetic efficacy. Further verification using other cells is required.

On the other hand, the change in liquidity shown using artificial membranes was < 1° in terms of change with temperature; therefore, we cannot conclusively state that the change in lipid bilayer fluidity is a key mechanism of anesthesia. Following a change in cell membrane fluidity, we would expect an amplification mechanism to ultimately achieve whole‐body anesthesia. Several reports suggest that lateral diffusion of receptor proteins influences signal transduction. Using quantum dots, Bannai *et al*. 21 showed that the GABA_A_ receptor that accumulates in the postsynaptic membrane in association with Ca influx (excitatory nerve activity) into the cell via the *N*‐nitrosodimethylamine (NMDA) receptor becomes easy to move on the cell membrane, and the number of receptors decreases with deviation inside the synapse. A decrease in the number of GABA_A_ receptors within the synapse causes attenuation of inhibitory neurotransmission. On the contrary, it was reported that the metabotropic glutamate receptor–phospholipase C‐to‐inositol trisphosphate receptor signaling pathway is necessary for the accumulation and stable maintenance of GABA_A_ receptors on synapses. In addition, Suzuki *et al*. 22, 23 reported that a temporary pause of about 0.5 s is necessary for the CD59 cluster, diffusing on the cell membrane, to transmit an intracellular signal. These findings suggest that when a signal is transferred into the cell, it is necessary for the receptor to remain on the cell membrane for a certain period of time.

Therefore, by increasing the fluidity of the membrane receptor protein, ISO is able to inhibit signal transduction. Because signal transduction involves multiple steps and maintains a balanced state, it is very difficult to identify the multiple active sites affected by volatile anesthetics in each system. The present study only evaluated the liquidity of GPI and TfR, and future studies are required to investigate changes in liquidity on exposure to anesthetics, with a focus on anesthetic‐related receptors such as GABA_A_, NMDA, and the metabotropic glutamate receptor.

In conclusion, our findings suggest that ISO is an anesthetic agent that affects the mobility of transmembrane protein molecules. This study is the first to use live cells to examine the mechanism of action of volatile anesthetics on cell membranes and membrane proteins in detail.

## Author contributions

JO and KK (co‐corresponding) planned the study and wrote the manuscript. SF performed most of the experiments. KK supervised FRAP analysis, and supervised and complemented the writing. SS designed HaloTag fusion proteins. KA and HK designed the experiment of GC/MS. GS provided comments pertaining to the manuscript.

## Supporting information


**Fig. S1**. FRAP analysis of HEK293T cells and neural cells. Influence of anesthetic on the recovery of fluorescence intensity in HEK293T cells (A) and neural cells (C). The *t*_1/2_ of fluorescence recovery rate of Alexa488‐GPI in HEK293T cells (B) and neural cells (D) treated with 1 mm ISO for 30 min or 60 μm MDZ for 30 min. Blue circle: control; orange circle: 1 mm ISO; gray circle: 60 μm MDZ.

**Fig. S2**. Whole‐plasmid sequences of pcDNA3‐TfRHL (A) and pcDNA3‐ssHLGPI (B).

## References

[1] Franks NP and Lieb WR (1987) Neuron membranes: anaesthetics on the mind. Nature 328, 113–114. 3600786 10.1038/328113a0

[2] Tsai YS , Ma SM , Kamaya H and Ueda I (1987) Fourier transform infrared studies on phospholipid hydration: phosphate‐oriented hydrogen bonding and its attenuation by volatile anesthetics. Mol Pharmacol 31, 623–630. 3600607

[3] Hamanaka T , Nakagawa T , Kito Y , Nishimura S , Uchida I and Mashimo T (1998) Binding of volatile anesthetics to purple membranes studied by X‐ray diffraction. Toxicol Lett 100–101, 397–403. 10.1016/s0378-4274(98)00213-6 10049171

[4] Tang P and Xu Y (2002) Large‐scale molecular dynamics simulations of general anesthetic effects on the ion channel in the fully hydrated membrane: the implication of molecular mechanisms of general anesthesia. Proc Natl Acad Sci USA 99, 16035–16040. 12438684 10.1073/pnas.252522299 PMC138560

[5] Yoshida T , Okabayashi H , Takahashi K and Ueda I (1984) A proton nuclear magnetic resonance study on the release of bound water by inhalation anesthetic in water‐in‐oil emulsion. Biochim Biophys Acta 772, 102–107. 6712949 10.1016/0005-2736(84)90522-4

[6] Ueda I , Hirakawa M , Arakawa K and Kamaya H (1986) Do anesthetics fluidize membranes? Anesthesiology 64, 67–71. 3942336 10.1097/00000542-198601000-00010

[7] Suzuki S , Kiyosue K , Hazama S , Ogura A , Kashihara M , Hara T , Koshimizu H and Kojima M (2007) Brain‐derived neurotrophic factor regulates cholesterol metabolism for synapse development. J Neurosci 27, 6417–6427. 17567802 10.1523/JNEUROSCI.0690-07.2007 PMC6672445

[8] McDougall SJ , Peters JH , LaBrant L , Wang X , Koop DR and Andresen MC (2008) Paired assessment of volatile anesthetic concentrations with synaptic actions recorded *in vitro* . PLoS ONE 3, e3372. 18841202 10.1371/journal.pone.0003372 PMC2556393

[9] Simon W , Hapfelmeier G , Kochs E , Zieglgansberger W and Rammes G (2001) Isoflurane blocks synaptic plasticity in the mouse hippocampus. Anesthesiology 94, 1058–1065. 11465598 10.1097/00000542-200106000-00021

[10] Jones MV , Brooks PA and Harrison NL (1992) Enhancement of gamma‐aminobutyric acid‐activated Cl‐ currents in cultured rat hippocampal neurones by three volatile anaesthetics. J Physiol 449, 279–293. 1326046 10.1113/jphysiol.1992.sp019086 PMC1176079

[11] Steward A , Allott PR , Cowles AL and Mapleson WW (1973) Solubility coefficients for inhaled anaesthetics for water, oil and biological media. Br J Anaesth 45, 282–293. 4573000 10.1093/bja/45.3.282

[12] Zhao S , Zhu Y , Xue R , Li Y , Lu H and Mi W (2012) Effect of midazolam on the proliferation of neural stem cells isolated from rat hippocampus. Neural Regen Res 7, 1475–1482. 25657682 10.3969/j.issn.1673-5374.2012.19.005 PMC4308778

[13] Nakahiro M , Yeh JZ , Brunner E and Narahashi T (1989) General anesthetics modulate GABA receptor channel complex in rat dorsal root ganglion neurons. FASEB J 3, 1850–1854. 2541038 10.1096/fasebj.3.7.2541038

[14] Kusumi A , Koyama‐Honda I and Suzuki K (2004) Molecular dynamics and interactions for creation of stimulation‐induced stabilized rafts from small unstable steady‐state rafts. Traffic 5, 213–230. 15030563 10.1111/j.1600-0854.2004.0178.x

[15] Lenne PF , Wawrezinieck L , Conchonaud F , Wurtz O , Boned A , Guo XJ , Rigneault H , He HT and Marguet D (2006) Dynamic molecular confinement in the plasma membrane by microdomains and the cytoskeleton meshwork. EMBO J 25, 3245–3256. 16858413 10.1038/sj.emboj.7601214 PMC1523176

[16] Platholi J , Herold KF , Hemmings HC Jr and Halpain S (2014) Isoflurane reversibly destabilizes hippocampal dendritic spines by an actin‐dependent mechanism. PLoS ONE 9, e102978. 25068870 10.1371/journal.pone.0102978 PMC4113311

[17] Lemkuil BP , Head BP , Pearn ML , Patel HH , Drummond JC and Patel PM (2011) Isoflurane neurotoxicity is mediated by p75NTR‐RhoA activation and actin depolymerization. Anesthesiology 114, 49–57. 21169791 10.1097/ALN.0b013e318201dcb3 PMC3037980

[18] Lunardi N , Hucklenbruch C , Latham JR , Scarpa J and Jevtovic‐Todorovic V (2011) Isoflurane impairs immature astroglia development *in vitro*: the role of actin cytoskeleton. J Neuropathol Exp Neurol 70, 281–291. 21412172 10.1097/NEN.0b013e31821284e9 PMC3063859

[19] Aureli M , Grassi S , Prioni S , Sonnino S and Prinetti A (2015) Lipid membrane domains in the brain. Biochim Biophys Acta 1851, 1006–1016. 25677824 10.1016/j.bbalip.2015.02.001

[20] Mapleson WW (1996) Effect of age on MAC in humans: a meta‐analysis. Br J Anaesth 76, 179–185. 8777094 10.1093/bja/76.2.179

[21] Bannai H , Levi S , Schweizer C , Inoue T , Launey T , Racine V , Sibarita JB , Mikoshiba K and Triller A (2009) Activity‐dependent tuning of inhibitory neurotransmission based on GABAAR diffusion dynamics. Neuron 62, 670–682. 19524526 10.1016/j.neuron.2009.04.023

[22] Suzuki KG , Fujiwara TK , Edidin M and Kusumi A (2007) Dynamic recruitment of phospholipase C gamma at transiently immobilized GPI‐anchored receptor clusters induces IP3‐Ca2+ signaling: single‐molecule tracking study 2. J Cell Biol 177, 731–742. 17517965 10.1083/jcb.200609175 PMC2064217

[23] Suzuki KG , Fujiwara TK , Sanematsu F , Iino R , Edidin M and Kusumi A (2007) GPI‐anchored receptor clusters transiently recruit Lyn and G alpha for temporary cluster immobilization and Lyn activation: single‐molecule tracking study 1. J Cell Biol 177, 717–730. 17517964 10.1083/jcb.200609174 PMC2064216

